# An Increase in the Omega-6/Omega-3 Fatty Acid Ratio Increases the Risk for Obesity

**DOI:** 10.3390/nu8030128

**Published:** 2016-03-02

**Authors:** Artemis P. Simopoulos

**Affiliations:** The Center for Genetics, Nutrition and Health, 4330 Klingle Street NW, Washington, DC 20016, USA; cgnh@bellatlantic.net; Tel.: +1-202-462-5062; Fax: +1-202-462-5241

**Keywords:** obesity, omega-6 and omega-3 essential fatty acids, omega-6 and omega-3 fatty acid ratio, eicosanoids, browning of adipose tissue, endocannabinoids, FTO (Fat Mass and Obesity-Associated) Gene

## Abstract

In the past three decades, total fat and saturated fat intake as a percentage of total calories has continuously decreased in Western diets, while the intake of omega-6 fatty acid increased and the omega-3 fatty acid decreased, resulting in a large increase in the omega-6/omega-3 ratio from 1:1 during evolution to 20:1 today or even higher. This change in the composition of fatty acids parallels a significant increase in the prevalence of overweight and obesity. Experimental studies have suggested that omega-6 and omega-3 fatty acids elicit divergent effects on body fat gain through mechanisms of adipogenesis, browning of adipose tissue, lipid homeostasis, brain-gut-adipose tissue axis, and most importantly systemic inflammation. Prospective studies clearly show an increase in the risk of obesity as the level of omega-6 fatty acids and the omega-6/omega-3 ratio increase in red blood cell (RBC) membrane phospholipids, whereas high omega-3 RBC membrane phospholipids decrease the risk of obesity. Recent studies in humans show that in addition to absolute amounts of omega-6 and omega-3 fatty acid intake, the omega-6/omega-3 ratio plays an important role in increasing the development of obesity via both AA eicosanoid metabolites and hyperactivity of the cannabinoid system, which can be reversed with increased intake of eicosapentaenoic acid (EPA) and docosahexaenoic acid (DHA). A balanced omega-6/omega-3 ratio is important for health and in the prevention and management of obesity.

## 1. Introduction

Obesity is a complex condition involving the dysregulation of several organ systems and molecular pathways, including adipose tissue, liver, pancreas, gastrointestinal tract, the microbiome, the central nervous system (CNS), and genetics. The role of the CNS in obesity is receiving more attention as obesity rates rise and treatments continue to fail. While the role of the hypothalamus in the regulation of appetite and food intake has long been recognized, the roles of the CNS reward systems are beginning to be examined as the role of environmental influences on energy balance are explored. Furthermore, omega-3 fatty acids hold great promise in the prevention and management of obesity.

**Figure 1:**
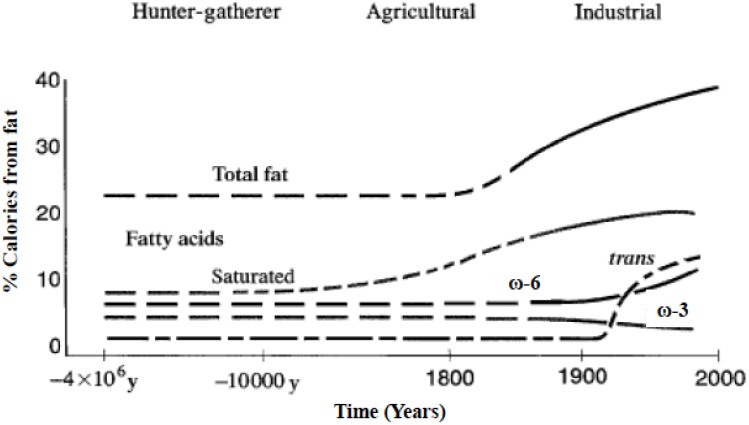
Hypothetical scheme of fat, fatty acid (ω6, ω3, trans, and total) intake (as percent of calories from fat). Data were extrapolated from cross-sectional analyses of contemporary hunter-gatherer populations and from longitudinal observations and their putative changes during the preceding 100 years (Modified from [3]).

Omega-6 and omega-3 polyunsaturated fatty acids (PUFAs) are essential fatty acids that must be derived from the diet, cannot be made by humans, and other mammals because of the lack of endogenous enzymes for omega-3 desaturation [1,2]. Due to agribusiness and modern agriculture western diets contain excessive levels of omega-6 PUFAs but very low levels of omega-3 PUFAs, leading to an unhealthy omega-6/omega-3 ratio of 20:1, instead of 1:1 that was during evolution in humans (Figure 1) [1,3].

**Figure 2:**
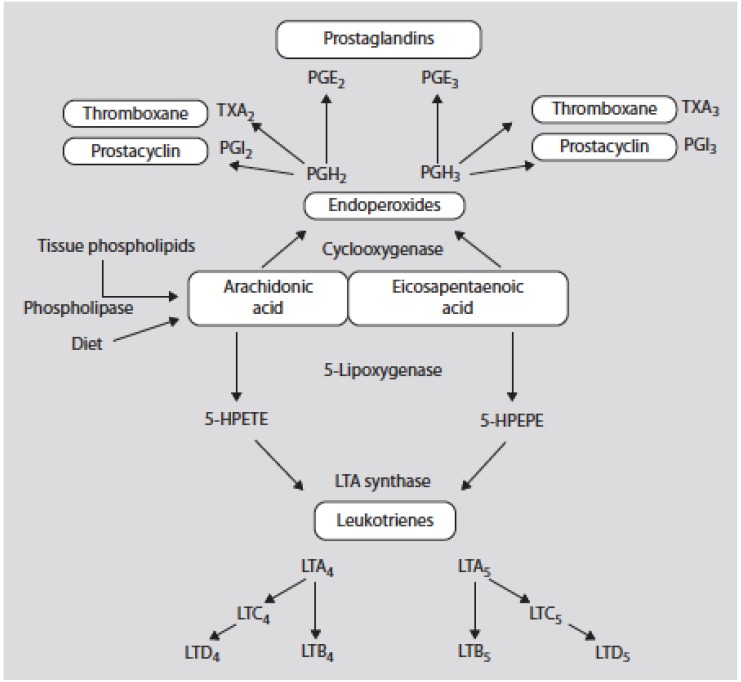
Oxidative metabolism of arachidonic acid (AA) and eicosapentaenoic acid by the cy-clooxygenase and 5-lipoxygenase pathways. 5-HPETE denotes the 5 hydroperoxyeicosatetranoic acid and 5-HPEPE denotes the 5-hydroxyeicosapentaenoic acid [3].

Eicosanoid products derived from omega-6 PUFAs (such as prostaglandin (PG) E2 and leukotriene (LT) B4 synthesized from arachidonic acid (AA)) are more potent mediators of thrombosis and inflammation than similar products derived from omega-3 PUFAs (PGE3 and LTB5 synthesized from eicosapentaenoic acid (EPA)) (Figure 2) (Table 1) [1,2,3].

Thus, an unbalanced omega-6/omega-3 ratio in favor of omega-6 PUFAs is highly prothrombotic and proinflammatory, which contributes to the prevalence of atherosclerosis, obesity, and diabetes [1,2,3,4,5,6]. In fact, regular consumption of diets rich in omega-3 PUFAs have been associated with low incidence of these diseases, particularly in Icelandic populations, Inuit indigenous people, and Native Americans in Alaska [7,8,9]. However, using fish oil as the primary source of omega-3 PUFAs to treat type 2 diabetes has not always met with success [6,10,11]. Although nutritional studies suggest that high omega-6/omega-3 ratios have contributed significantly to the “obesity epidemic” [12,13], clinical trials using omega-3 PUFAs as weight-reducing agents have produced conflicting findings of both positive [14,15,16] and negative effects [17,18,19] due to many factors (Table 2).

This paper focuses, on the differential aspects of omega-6 and omega-3 fatty acids and their ratio, in energy balance and in the prevention and management of obesity.

## 2. The Importance of the Omega-6/Omega-3 Fatty Acid Ratio: Metabolic, Physiological and Evolutionary Aspects

**Figure 3:**
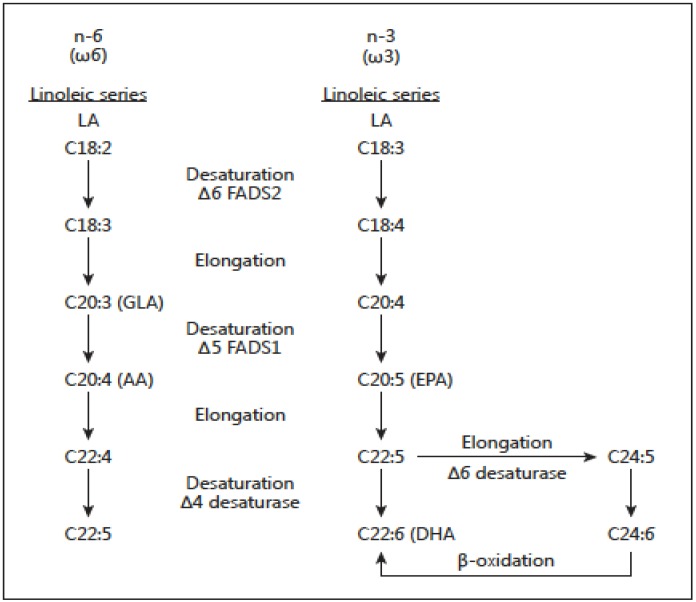
Desaturation and elongation of ω-3 and ω-6 fatty acids by the enzymes fatty acid de-saturases FADS2 (D6) and FADS1 (D5).

There are two classes of essential fatty acids (EFA), omega-6 and omega-3. The distinction between omega-6 and omega-3 fatty acids is based on the location of the first double bond, counting from the methyl end of the fatty acid molecule. Omega-6 fatty acids are represented by linoleic acid (LA) (18:2ω-6) and omega-3 fatty acids by alpha-linolenic acid (ALA) (18:3ω-3). LA is plentiful in nature and is found in the seeds of most plants except for coconut, cocoa, and palm. ALA, on the other hand, is found in the chloroplasts of green leafy vegetables, and in the seeds of flax, rape, chia, perilla and walnuts. Both essential fatty acids are metabolized to longer-chain fatty acids of 20 and 22 carbon atoms. LA is metabolized to arachidonic acid (AA) (20:4ω6) while ALA is metabolized to eicosapentaenoic acid (EPA) (20:5ω3) and docosahexaenoic acid (DHA) (22:6ω3). This is achieved by increasing the chain length and the degree of unsaturation by adding extra double bonds to the carboxyl end of the fatty acid molecule [20] (Figure 3). AA is found predominantly in the phospholipids of grain-fed animals, dairy and eggs. EPA and DHA are found in the oils of fish, particularly fatty fish.

In mammals, including humans, the cerebral cortex, retina, testis and sperm are particularly rich in DHA. DHA is one of the most abundant components of the brain’s structural lipids. DHA, like EPA, can be derived only from direct ingestion or by synthesis from dietary EPA or ALA: humans and other mammals, except for certain carnivores such as lions, can convert LA to AA and ALA to EPA and DHA, although the process is slow [21,22]. There is competition between omega-6 and omega-3 fatty acids for the desaturation enzymes. Both fatty acid desaturase 1 (FADS1) and fatty acid desaturase 2 (FADS2) prefer ALA to LA [21,23,24]. However a high LA intake, such as that characterizing Western diets, interferes with the desaturation and elongation of ALA [22,23,24,25]. Similarly, trans fatty acids interfere with the desaturation and elongation of both LA and ALA.

There are important genetic variables in fatty acid biosynthesis involving FADS1 and FADS2, which encode rate-limiting enzymes for fatty acid metabolism (Figure 3). Ameur *et al.* [26] performed genome-wide genotyping (*n =* 5652 individuals) of the FADS region in five European population cohorts and analyzed available genomic data from human populations, archaic hominins, and more distant primates. Their results show that present-day humans have two common FADS haplotypes A and D that differ dramatically in their ability to generate long-chain polyunsaturated fatty acids (LC-PUFAs). The most common haplotype, denoted haplotype D, was associated with high blood lipid levels (*p* = 1 × 10^−65^), whereas the less common haplotype (haplotype A) was associated with low blood lipid levels (*p* = 1 × 10^−52^). The haplotype D associated with the enhanced ability to produce AA and EPA from their precursors LA and ALA respectively is specific to humans and has appeared after the split of the common ancestor of humans and Neanderthals. This haplotype shows evidence of a positive selection in African populations in which it is presently almost fixed and it is less frequent outside of Africa. Haplotype D provides a more efficient synthesis of LC-PUFAs and in today’s high LA omega-6 dietary intake from vegetable oils, it leads to increased synthesis of AA from LA. As a result Haplotype D represents a risk factor for coronary heart disease (CHD), cancer, obesity, diabetes and the metabolic syndrome, adding further to health disparities in populations of African origin living in the West, in addition to lower socioeconomic status [27,28]. Furthermore, FADS2 is the limiting enzyme and there is some evidence that it decreases with age [21]. Premature infants [29], hypertensive individuals [30], and some diabetics [31] are limited in their ability to make EPA and DHA from ALA. These findings are important and need to be considered when making dietary recommendations. Genetic variants in FADS cluster are determinants of long-chain PUFA levels in circulation, cells and tissues. These genetic variants have been studied in terms of ancestry, and the evidence is robust relative to ethnicity. Thus, 80% of African Americans and about 45% of European Americans carry two copies of the alleles associated with increased levels of AA. It is quite probable that gene PUFA interactions induced by the modern Western diet are differentially driving the risk of diseases of inflammation (obesity, diabetes, atherosclerosis and cancer) in diverse populations.

As mentioned earlier, mammalian cells cannot convert omega-6 to omega-3 fatty acids because they lack the converting enzyme, omega-3 desaturase. Omega-6 and omega-3 fatty acids are not interconvertible, are metabolically and functionally distinct, and often have important opposing physiological effects, therefore their balance in the diet is important. When humans ingest fish or fish oil, the EPA and DHA from the diet partially replace the omega-6 fatty acids, especially AA, in the membranes of probably all cells, but especially in the membranes of platelets, erythrocytes, neutrophils, monocytes, and liver cells (reviewed in [3,32]). AA and EPA are the parent compounds for eicosanoid production. Because of the increased amounts of omega-6 in the Western diet, the eicosanoid metabolic products from AA, specifically prostaglandins, thromboxanes, leukotrienes, hydroxy fatty acids, and lipoxins, are formed in larger quantities than those derived from omega-3 fatty acids, specifically EPA [3]. The eicosanoids from AA are biologically active in very small quantities and, if they are formed in large amounts, they contribute to the formation of thrombus and atheromas; to allergic and inflammatory disorders, particularly in susceptible people; and to proliferation of cells [33]. Thus, a diet rich in omega-6 fatty acids shifts the physiological state to one that is proinflammatory, prothrombotic, and proaggregatory, with increases in blood viscosity, vasospasm, vasoconstriction and cell proliferation.

A balance existed between omega-6 and omega-3 fatty acids during the long evolutionary history of the genus Homo [34]. During evolution, omega-3 fatty acids were found in all foods consumed: particularly meat, fish, wild plants, nuts and berries [34,35,36,37,38,39,40,41,42,43,44,45,46,47,48,49,50]. Recent studies by Cordain *et al.* [51] on the composition of the meat of wild animals confirm the original observations of Crawford and Sinclair *et al.* [36,52]. However, rapid dietary changes over short periods of time as have occurred over the past 100–150 years is a totally new phenomenon in human evolution (Figure 1). A balance between the omega-6 and omega-3 fatty acids is a physiological state that is less inflammatory in terms of gene expression [53], prostaglandin and leukotriene metabolism, and interleukin-1 (IL-1) production [3].

Modern agriculture, by changing animal feeds as a result of its emphasis on production, has decreased the omega-3 fatty acid content in many foods: animal meats, eggs, and even fish [39,40,41,42]. Foods from edible wild plants contain a good balance of omega-6 and omega-3 fatty acids. Purslane, a wild plant, in comparison to spinach, red leaf lettuce, buttercrunch lettuce and mustard greens, has eight times more ALA than the cultivated plants [46]. Modern aquaculture produces fish that contain less omega-3 fatty acids than do fish grown naturally in the ocean, rivers and lakes [41]. The fatty acid composition of egg yolk from free-ranging chicken has an omega-6:omega-3 ratio of 1.3 whereas the United States Department of Agriculture (USDA) egg has a ratio of 19.9 [42]. By enriching the chicken feed with fishmeal or flaxseed, the ratio of omega-6:omega-3 decreased to 6.6 and 1.6 respectively.

Although diets differ in the various geographic areas [54], a number of investigators including Crawford [36], Cordain [51], Eaton [55] and Kupiers [56] have shown that during the Paleolithic period, the diets of humans included equal amounts of omega-6 and omega-3 fatty acids from both plants (LA + ALA) and from the fat of animals in the wild and fish (AA + EPA + DHA). Recent studies by Kuipers *et al.* [56] estimated macronutrient and fatty acid intakes from an East African Paleolithic diet in order to reconstruct multiple Paleolithic diets and thus estimate the ranges of nutrient intakes on which humans evolved. They found (range of medians in energy %) intakes of moderate-to-high protein (25%–29%), moderate-to-high fat (30%–39%) and moderate carbohydrates (39%–40%). Just as others have concluded previously, Kuipers *et al.* [56] stated, “compared with Western diets, Paleolithic diets contained consistently high protein and long-chain PUFA and lower LA”. Guil-Guerrero J *et al.* [57] determined the fat composition of frozen mammoths (from 41,000 to 34,000 years BP), Bisons from early Holocene (8200–9300 years BP) and horses from middle Holocene (4600–4400 years BP), often consumed by Paleolithic/Neolithic hunters gatherers, and concluded, that “the animal fat contained suitable amounts of omega-3 and omega-6 fatty acids, possibly in quantities sufficient to meet today’s dietary requirements for good health”. The elucidation of sources of omega-3 fatty acids available for the humans who lived in the Paleolithic and Neolithic is highly relevant to ascertain the availability of nutrients at that time and to draw conclusions about healthy dietary habits for present-day humans. As in previous studies, the amount of ALA was higher than LA in the fat of the frozen specimens [58,59] (Table 3 and Table 4).

## 3. Effects of Omega-6 and Omega-3 Fatty Acids and their Ratio on Obesity

Experimental studies have suggested that omega-3 and omega-6 fatty acids may elicit divergent effects on body fat gain through mechanisms of adipogenesis [60], lipid homeostasis [61,62], brain-gut-adipose tissue axis [63], and systemic inflammation [64]. Metabolites of AA (20:4ω-6) play important roles in the terminal differentiation of pre-adipocyte to mature adipocyte [65]. This effect can be inhibited by omega-3 fatty acids at multiple steps [66,67,68,69]. Omega-6 fatty acids increase cellular triglyceride content by increasing membrane permeability [70], while omega-3 fatty acids reduce fat deposition in adipose tissues by suppressing lipogenic enzymes and increasing β-oxidation [71]. In addition, omega-6 and omega-3 fatty acids differentially modulate the brain-gut-adipose tissue axis [63] and the inflammatory properties of downstream eicosanoids, which ultimately affect pre-adipocyte differentiation and fat mass growth [72]. White adipocytes are storing energy in the form of triglycerides whereas brown adipocytes dissipate energy from triglycerides by producing heat (Table 5). In rodents and possibly in humans both types of fat cells participate in the total energy balance. By altering rates of adipocyte differentiation and proliferation, differences in fatty acid composition of dietary fats may also contribute to adipose tissue development, in particular with respect to the relative intake of omega-6 and omega-3 fatty acids. The omega-6/omega-3 ratio determines the availability of omega-6-AA within adipose tissue and thus the level of various prostaglandins derived from the cyclooxygenase-mediated pathways, which can be blocked by omega-3 fatty acids (Figure 2). Recent studies have shown that perinatal exposure of mice to a high omega-6 fatty acid diet (similar to Western diet) results in a progressive accumulation of body fat across generations, which is consistent with the fact that in humans, overweight and obesity have steadily increased in the last decades, and emerge earlier in life [12,73]. Furthermore, AA metabolites prostaglandins E2 and F2α have an inhibitory role in the browning process of white fat cells converted into energy-dissipating brown fat cells which are believed to play a role in controlling energy balance by lowering body weight [74,75,76,77,78,79,80,81,82] (Table 5).

High intake of omega-6 fatty acids during the perinatal period is associated with increased adiposity in the offspring. In human studies the level of AA in adipose tissue is associated with the BMI and overweight status of children. High omega-6/omega-3 fatty acids in umbilical cord red blood cell (RBC) membrane phospholipids was associated with high subscapular skin-fold thickness at 3 years of age [5].

Animal and human studies have shown that EPA and DHA supplementation may be protective against obesity, and may reduce weight gain in already obese animals and humans [83]. Specifically, studies demonstrated a reduction in visceral (epididymal and/or retroperitoneal) fat in rats fed high lipid diets that incorporate omega-3 PUFAs [75,78,79,84,85,86,87], and the effect was dose-dependent [85]. The reduction in visceral fat was associated with a decrease in adipocyte size [85,86] and number [87]. High fat diets rich in omega-6 fatty acids have been shown to increase the risk of leptin resistance, diabetes, and obesity in humans and rodents [76,88]. AA impairs hypothalamic leptin signaling and energy homeostasis in mice [77]. The inhibitory role of AA has been suggested in both basal and insulin-stimulated leptin expression and production [88].

### 3.1. The Fat-1 Transgenic Mouse Model

Many of the problems involving dietary animal studies can be overcome by the fat-1 transgenic mouse model that carries a Caenorhabditis elegans fat-1 gene encoding an omega-3 fatty acid desaturase. This enzyme can convert omega-6 to omega-3 fatty acids by adding a double bond at the omega-3 position [89]. Consequently, fat-1 not only increases the levels of omega-3 fatty acids but also concomitantly decreases omega-6 fatty acids as well as the omega-6/omega-3 ratio—a goal difficult to achieve through dietary means alone, yet important for health benefits. Although not fully equated to a dietary approach, the transgenic model produces the same types of omega-3 fatty acids as those obtained through diet [89].

Furthermore, the fat-1 model offers numerous advantages in the studies of the health benefits of omega-3 fatty acids, because the transgenic model allows elucidation of the mechanisms of actions of omega-3 fatty acids without the confounding issues associated with dietary approaches, such as dose, composition, and duration of treatment applied in different studies [16,90,91] (Table 2). The use of fat-1 mouse model avoids these concerns by feeding exactly the same diet to the transgenic and wild type (WT) mice. Because FAT-1 is an enzyme, the production of omega-3 fatty acids in mice is limited by the amount of available substrate: omega-6 fatty acids. The degree of increase in omega-3 fatty acids (3- to 4-fold) required to improve metabolic parameters in fat-1 transgenic mice has been shown to be achievable through dietary means in animals and humans [92]. Recently Li *et al.* [93] carried out a comprehensive study using the fat-1 model to better define how alterations in the tissue levels of omega-6 and omega-3 fatty acids affect energy balance, lipid and glucose metabolism, chronic inflammation, and the underlying molecular events (or mechanisms). Their results show that when challenged with high-fat diets, fat-1 mice strongly resisted obesity, diabetes, hypercholesterolemia, and hepatic steatosis. Endogenous elevation of omega-3 PUFAs and reduction of omega-6 PUFAs did not alter the amount of food intake but led to increased energy expenditure in the fat-1 mice. These metabolic phenotypes were accompanied by attenuation of the inflammatory state because tissue levels of prostaglandin E2, leukotriene B4, monocyte chemoattractant protein-1, and TNF-α were significantly decreased. The TNF-α-induced NF-κB signaling was almost completely abolished. Consistent with the reduction in chronic inflammation and a significant increase in peroxisome proliferator-activated receptor-γ activity in the fat-1 liver tissue, hepatic insulin signaling was sharply elevated. The activities of prolipogenic regulators, such as liver X receptor, stearoylCoA desaturase-1, and sterol regulatory element binding protein-1 were sharply decreased, whereas the activity of peroxisome proliferator-activated receptor-α, a nuclear receptor that facilitates lipid β-oxidation, was markedly increased [93]. Thus, endogenous conversion of omega-6 to omega-3 PUFAs via fat-1 strongly protects against obesity, diabetes, inflammation, and dyslipidemia and may represent a novel therapeutic modality to treat these prevalent disorders.

### 3.2. Human Studies

Randomized controlled trials in humans examining the relationship between omega-3 supplementation and body composition have produced conflicting results due to many factors summarized in Table 2 [19,83]. This may be due to differences in study design, dosage, not taking into consideration the omega-6/omega-3 ratio of the background diet, timing, and duration of omega-3 PUFA administration, use of other supplements in addition to omega-3 PUFA, and demographics of the study population. Furthermore, in many studies the determination of omega-6/omega-3 fatty acids was based on food frequency questionnaires, which are not as accurate as direct measurements of fatty acids in RBC membrane phospholipids. Several studies have provided supporting evidence for a role of omega-3 PUFAs in body composition [94], weight reduction [95], less hunger and more fullness [96]. These findings support a potential role for omega-3 in appetite regulation in humans. Some intervention studies showed that omega-3 fatty acid supplementation reduced body weight and obesity in lean [14], overweight [95,97] and obese [98] individuals. Couet *et al.* [14] noted a 22% increase in basal lipid oxidation with 6 grams of fish oil for 3 weeks. Omega-3 fatty acids are long term metabolic fuel partitioners with greater partitioning towards β-oxidation in men than in women.

Omega-6 and omega-3 red blood cell membrane phospholipid determinations represent biomarkers of dietary intake plus endogenous metabolism and represent the most accurate way to carry out preventive studies and clinical intervention studies to evaluate their role in weight gain and obesity [99]. Wang *et al.* [100] conducted a prospective analysis to examine the association of baseline red blood cell membrane phospholipids of omega-3 fatty acids, omega-6 fatty acids, omega-6/omega-3 ratio and trans fatty acids with the longitudinal changes in body weight and the risk of becoming overweight or obese during a mean of 10.4 years follow up in the NIH Women’s Health Initiative Study. The results of this prospective study showed that baseline red blood cell membrane phospholipids cis omega-3 fatty acids is inversely associated, and cis omega-6 fatty acids are positively associated, with longitudinal weight gain in initially normal weight healthy women. This is the first study to prospectively examine omega-3 and omega-6 fatty acids in red blood cell membrane phospholipids in relation to weight gain and the risk of becoming overweight or obese. After multivariable adjustment, significant positive associations with weight gain were found only for dihomo-γ-linolenic acid (DGLA), LA, and Gamma-linolenic acid (GLA) among omega-6 fatty acids and trans 18:1 among trans fatty acids; while inverse associations were found with EPA among omega-3 fatty acids. The authors state that, “the variations by individual fatty acids may be due to unknown and uncontrolled factors involved in the conversion and metabolism of each fatty acid, and should be interpreted cautiously given the multiple comparisons. This study included only women who had normal BMI at baseline to minimize potential confounding and address the risk of becoming overweight or obese”. To further evaluate the impact of baseline BMI on the results, Wang *et al.* [100] stratified analyses by baseline BMI levels (18.5–≤23, 23–≤25 kg/m^2^) and also included women who were already overweight or obese at baseline (baseline BMI ≥ 25 kg/m^2^) in sensitivity analyses. Similar patterns of associations were found in these additional analyses. In conclusion, this prospective study provided strong suggestive evidence that omega-3 fatty acids in RBC membrane phospholipids are inversely associated, while cis omega-6 fatty acids, omega-6/omega-3 ratio, and trans fatty acids are positively associated, with longitudinal weight gain.

## 4. Genetics: The Fat Mass and Obesity-Associated Gene

Genome-Wide Association Studies (GWAS) have identified more than 90 loci that contain genetic variants associated with obesity. Many of these variants are in intronic regions. The strongest genetic association with risk to polygenic obesity are single-nucleotide variants (SNV) in intron 1 and 2 of the FTO (fat mass and obesity associated) gene. There are 89 SNVs in FTO intron 1 and 2. Deciphering how these variants regulate gene expression has been difficult. Recently Claussnitzer *et al.* [101] reported a strategy and defined the causal SNV and the mechanisms of function in preadipocytes. The authors provide evidence for the rs 1421085 T to C SNV to result in a cellular phenotype consistent with obesity in primary human adipocytes, including decreased mitochondrial energy generation and increased triglyceride accumulation.

Their study provided evidence that the risk allele rs 1411085 T to C SNV resulted in increased expression of IRX3 and IRX5 genes in pre-adipocytes, which shifted the development of these cells toward the “white program” and increased lipid storage, whereas knockdown of IRX3 and IRX5 genes restored thermogenesis in adipocytes from persons at high risk for obesity. Thus, the risk allele functioned similarly to AA metabolites, PGI2 and PGF2a, increasing proliferation of white adipose tissue and decreasing its browning respectively, whereas the knockdown of IRX3 and IRX5 genes functioned similarly to omega-3 fatty acid metabolites increasing the browning of the adipose tissue, mitochondrial biogenesis and thermogenesis. The arachidonic acid metabolites PGI2 and PGF2a lead to increases in white adipose tissue and decreases its browning respectively. Human studies have shown a direct relationship between plasma arachidonic acid levels and infant body weight, as well as between AA levels in adipose tissue lipids and BMI in children in Cyprus and Crete [102,103]. AA directly inhibits UCPI gene expression. Considering the high omega-6/omega-3 fatty acid ratio of Western diets and the role of AA in adipose cell differentiation, proliferation, and decreasing browning of white adipose tissue, further research should include studies on the effects of omega-3 fatty acids in blocking the effects of the risk allele (rs 1421085), which appears to be responsible for the association between the first intron of FTO gene and obesity in humans.

## 5. Omega-6/Omega-3 Fatty Acid Ratio: Endocannabinoid System

A diet high in the omega-6/omega-3 ratio causes an increase in the endocannabinoid signaling and related mediators, which lead to an increased inflammatory state, energy homeostasis, and mood. In animal experiments a high omega-6 acid intake leads to decreased insulin sensitivity in muscle and promotes fat accumulation in adipose tissue. Nutritional approaches with dietary omega-3 fatty acids reverse the dysregulation of this system, improve insulin sensitivity and control body fat.

Endocannabinoids are lipids, derived from the omega-6 AA. Their concentrations are regulated by (1) dietary intake of omega-6 and omega-3 fatty acids; and (2) by the activity of biosynthetic and catabolic enzymes involved in the endocannabinoid pathway, which is an important player in regulation of appetite and metabolism [81,104]. The endocannabinoid system is involved in regulation of energy balance and sustained hyperactivity of the endocannabinoid system contributes to obesity [81,82]. AA is the precursor of 2-arachidonoylglycerol (2-AG) and anandamide (AEA). Increasing the precursor pool of AA causes excessive endocannabinoid signaling leading to weight gain and a metabolic profile associated with obesity. Endocannabinoids activate endogenous cannabinoid CB1 and CB2 receptors in the brain, liver, adipose tissue, and the gastrointestinal tract [105]. Activation of CB1 receptors in the hypothalamus leads to increased appetite and food intake [82]. In mouse experiments endocannabinoids selectively enhance sweet taste, which in the current highly palatable food supply stimulate food intake [106]. The endocannabinoid system functions in concert with other systems regulating food intake and energy balance, and is regulated by leptin, insulin, ghrelin, cholecystokinin, and other signals. Targeting the endocannabinoid system has been a strategy for weight loss. Randomized controlled clinical trials in overweight or obese humans showed that CB1 receptor antagonists such as rimonabant led to significant weight loss after one year of treatment [107]. However the drug was withdrawn from the market due to severe side effects that led to increased risk of anxiety, depression, and suicide [108].

Alvheim *et al.* [80] carried out an experiment in mice at six weeks of age, in which increasing the linoleic acid in the diet led to increases in AA in red blood cell membrane phospholipids, elevated 2-AG and AEA in liver, elevated plasma-leptin and resulted in larger adipocytes and more macrophage infiltration in adipose tissue. It was also noted that a higher linoleic acid increased feed efficacy and caused greater weight gain than isocaloric diets containing less LA. Increasing the dietary LA from 1% to 8% of energy increased liver endocannabinoid levels, which increased the risk of developing obesity, even in a low fat diet. Mice chronically deficient in omega-3 PUFA have significantly lower concentrations of DHA in brain phospholipids, and higher 2-AG (derived from AA) compared to mice with sufficient omega-3 PUFA in their diet [82]. Furthermore, omega-3 PUFA supplementation of mice with 10% weight DHA-rich fish oil for 4 weeks led to higher brain DHA levels compared to mice on a low omega-3 PUFA diet, and led to a significant decrease in brain 2-AG and brain AA. This nutritional approach with dietary omega-3 PUFA, reversed the dysregulation of the cannabinoid system, improved insulin sensitivity and decreased central body fat.

## 6. Conclusions and Recommendations

- Human beings evolved on a diet that was balanced in the omega-6 and omega-3 essential fatty acids.
- A high omega-6 fatty acid intake and a high omega-6/omega-3 ratio are associated with weight gain in both animal and human studies, whereas a high omega-3 fatty acid intake decreases the risk for weight gain. Lowering the LA/ALA ratio in animals prevents overweight and obesity.
- Omega-6/omega-3 fatty acids compete for their biosynthetic enzymes and because they have distinct physiological and metabolic properties, their balanced omega-6/omega-3 ratio is a critical factor for health throughout the life cycle.
- Adipose tissue is the main peripheral target organ handling fatty acids, and AA is required for adipocyte differentiation (adipogenesis). The increased LA and AA content of foods has been accompanied by a significant increase in the AA/EPA + DHA ratio within adipose tissue, leading to increased production in AA metabolites, PGI2 which stimulates white adipogenesis and PGF2α which inhibits the browning process, whereas increased consumption of EPA and DHA leads to adipose tissue homeostasis through adipose tissue loss and increased mitochondrial biogenesis.
- High omega-6 fatty acid intake leads to hyperactivity of endocannabinoid system, whereas omega-3 fatty acids lead to normal homeostasis (decrease hyperactivity).
- High omega-6 fatty acids increase leptin resistance and insulin resistance, whereas omega-3 fatty acids lead to homeostasis and weight loss.
- Because a high omega-6/omega-3 ratio is associated with overweight/obesity, whereas a balanced ratio decreases obesity and weight gain, it is essential that every effort is made to decrease the omega-6 fatty acids in the diet, while increasing the omega-3 fatty acid intake. This can be accomplished by (1) changing dietary vegetable oils high in omega-6 fatty acids (corn oil, sunflower, safflower, cottonseed, and soybean oils) to oils high in omega-3s (flax, perilla, chia, rapeseed), and high in monounsaturated oils such as olive oil, macadamia nut oil, hazelnut oil, or the new high monounsaturated sunflower oil; and (2) increasing fish intake to 2–3 times per week, while decreasing meat intake.
- In clinical investigations and intervention trials it is essential that the background diet is precisely defined in terms of the omega-6 and omega-3 fatty acid content. Because the final concentrations of omega-6 and omega-3 fatty acids are determined by both dietary intake and endogenous metabolism, it is essential that in all clinical investigations and intervention trials the omega-6 and omega-3 fatty acids are precisely determined in the red blood cell membrane phospholipids. In severe obesity drugs and bariatric surgery have been part of treatment.
- The risk allele rs 1421085 T to C SNV in intron 1 and 2 in the FTO gene functioned similarly to AA metabolites, PGI2 and PGF2a increasing proliferation of white adipose tissue and decreasing its browning respectively, whereas the knockdown of IRX3 and IRX5 genes functioned similarly to omega-3 fatty acid metabolites increasing the browning of white adipose tissue, mitochondrial biogenesis, and thermogenesis. Therefore, further research should include studies on the effects of omega-3 fatty acids in blocking the effects of the risk allele (rs 1421085), which appears to be responsible for the association between the first intron of FTO gene and obesity in humans.
- In the future studies on genetic variants from GWAS will provide opportunities to precisely treat and prevent obesity by both nutritional and pharmaceutical interventions.

Obesity is a preventable disease that can be treated through proper diet and exercise. A balanced omega-6/omega-3 ratio 1–2/1 is one of the most important dietary factors in the prevention of obesity, along with physical activity. A lower omega-6/omega-3 ratio should be considered in the management of obesity.

## Figures and Tables

**Table 1:** Effects of Ingestion of eicosapentaenoic acid (EPA) and docosahexaenoic acid (DHA) from Fish or Fish Oil. Modified from [3].

− Decreased production of prostaglandin E2 (PGE2) metabolites
− A decrease in thromboxane A2, a potent platelet aggregator and vasoconstrictor
− A decrease in leukotriene B4 formation, an inducer of inflammation, and a powerful inducer of leukocyte chemotaxis and adherence
− An increase in thromboxane A3, a weak platelet aggregator and weak vasoconstrictor
− An increase in prostacyclin PGI3
− Both PGI2 and PGI3 are active vasodilators and inhibitors of platelet aggregation
− An increase in leukotriene B5, a weak inducer of inflammation and a weak chemotactic agent

**Table 2:** Factors that affect outcomes in Obesity studies leading to conflicting results in clinical intervention trials (Modified from Reference [19]).

- Determination of the composition of the background diet in terms of omega-6 and omega-3 fatty acids and inflammatory markers *i.e.*, US, UK and Northern European countries have the highest amount of LA + AA in their diets, which competes with omega-3 PUFAs; they also have the lowest amount of vegetable and fruit intake, which are needed for optimal absorption of omega-3 PUFA from supplements
- Background inflammation
- Some studies are using fish and others omega-3 supplements; studies show that a continuous daily intake of omega-3 supplements leads to higher concentrations in the blood than eating fish two times/week
- Variation in the dose of omega-3 fatty acids
- Variation in the number of subjects
- Variation in the severity of disease
- Variation in the pharmacologic treatment
- Genetic variants predisposing to Obesity
- Dietary intake by means of questionnaires instead of actual measurements of omega-3 PUFAs in the red blood cell membrane phospholipids or plasma is a major problem that leads to conflicting results
- Length of intervention
- Genetic variants in the metabolism of omega-6 and omega-3 fatty acids

**Table 3:** Estimated Omega-3 and Omega-6 Fatty Acid intake in the Late Paleolithic Period (g/day) ^a,b,c^.

**Plants**	
LA	4.28
ALA	11.40
**Animals**	
LA	4.56
ALA	1.21
**Total**	
LA	8.84
ALA	12.60
**Animal**	
AA (ω6)	1.81
EPA (ω3)	0.39
DTA (ω6)	0.12
DPA (ω3)	0.42
DHA (ω3)	0.27
**Ratios of ω6/ω3**	
LA/ALA	0.70
AA + DTA/EPA + DPA + DHA	1.79
Total ω6/ω3	0.79 ^b^

^a^ Data from Eaton *et al.* [58]; ^b^ Assuming an energy intake of 35:65 of animal: plant sources; ^c^ LA, linoleic acid; ALA, linolenic acid; AA, arachidonic acid; EPA, eicosapentaenoic acid; DTA, docosatetranoic acid; DPA, docosapentaenoic acid; DHA, docosahexaenoic acid.

**Table 4:** Omega-6/Omega-3 Ratios in Different Populations.

Population	ω-6/ω-3
Paleolithic	0.79
Greece prior to 1960	1.00–2.00
Current Japan	4.00
Current India, rural	5–6.1
Current UK and northern Europe	15.00
Current US	16.74
Current India, urban	38–50

**Table 5:** Opposing Effects of Omega-6 and Omega-3 Fatty Acids in Obesity.

Conditions	Omega-6	Omega-3
Adipogenesis (Pre-adipocyte-Adipocyte)	High AA via the PI2 receptor activates the cAMP protein kinase A, signaling pathway leads to proliferation and differentiation of WAT, prevention of its browning through inhibition of PPARy target genes including UCPI, decrease mitochondrial biogenesis [74], increasing triglycerides [73], insulin resistance, leptin resistance, decreased adiponectin levels, decreased fatty acid oxidation and hepatic steatosis [4].	High EPA and DHA partially inhibit cAMP signaling pathways triggered by AA at levels upstream of PKA [71] block COX-2 metabolites PGI2 and PGEF2a that stimulate white adipogenesis and inhibit the browning process respectively, prevent increased triglycerides and adipose tissue proliferation through UCP-I and PPARy activation, increased mitochondrial biogenesis, increased fatty acid oxidation, and apoptosis [71,75].
Inflammation	AA metabolites prostaglandin 2 thromboxane 2 and leukotriene 4 are prothrombotic and proinflammatory leading to increased production of IL-1, IL-6, NFKB and TNF and inflammation [1,64].	High dietary intake of EPA and DHA blocks the metabolites of AA and prevents inflammation, which is the hallmark of obesity [1,64,67].
Insulin Resistance Leptin Resistance Adiponectin	AA leads to insulin resistance, leptin resistance, lower adiponectin and hepatic steatosis. AA blunts PI3-Akt pathway leading to leptin resistance in the brain and deregulation of food intake [19,76,77].	EPA and DHA regulate glucose utilization, insulin sensitivity (Akt phosphorylation) in part mediated by PPARy and AMPK activation [19]. EPA and DHA regulate the secretion of adipokines involved in energy homeostasis and intermediate metabolism and in glucose and lipid metabolism. DHA restores insulin sensitivity in skeletal muscle by preventing lipotoxicity and inflammation [78,79].
Cannabinoids	AA increases the concentration of (2-AG) and (AEA) leading to excessive endocannabinoid signaling, and dysregulation of the cannabinoid system, weight gain, larger adipocytes and more macrophages in adipose tissue [80], inflammation and a metabolic profile associated with obesity [81,82].	EPA and DHA decrease 2-AG and AA in the brain while increasing DHA, decreasing the dysregulation of the cannabinoid system, improving insulin sensitivity and decreasing central body fat.
